# Statistical optimization of culture medium for production of exopolysaccharide from endophytic fungus Bionectria ochroleuca and its antitumor effect in vitro

**DOI:** 10.17179/excli2016-154

**Published:** 2016-03-11

**Authors:** Yun Li, Shoujun Guo, Hui Zhu

**Affiliations:** 1 School of Life Sciences and Food Technology, Hanshan Normal University, Chaozhou, Guangdong 521041, China

**Keywords:** endophytic fungi, Bionectria ochroleuca, exopolysaccharide, antitumor activity

## Abstract

Endophytic fungi have been recognized as possible useful sources of bioactive metabolites. However, exopolysaccharide (EPS) production from endophytic fungi and its antitumor activity have been less explored. In the present study, endophtic fungus *Bionectria ochroleuca* M21 was exploited for the production of EPS in submerged culture. Among tested medium components, glucose, yeast extract, MgSO_4_ and Tween80 were found to be effective and significant on EPS production. Response surface methodology (RSM) was employed to optimize medium composition. The results showed that the significant factors were glucose, yeast extract and Tween80. The optimal medium was observed at the composition of glucose 55.7 g/L, yeast extract 6.04 g/L, MgSO_4_ 0.25g/L and Tween80 0.1 % (v/v). Using the optimized medium, EPS production was achieve at 2.65 ± 0.16 g/L after 4 days fermentation in a 5L bioreactor. Examination of cytotoxicity showed that the EPS from *B. ochroleuca* M21 did not have cytotoxic activity on human liver HL-7702 cells at concentration 0.025-1.6 mg/mL. In contrast, the EPS exhibited antiproliferative activities against cell lines of liver cancer (HepG2), gastric cancer (SGC-7901) and colon cancer (HT29) in a dose- and time-dependent manner in the concentration ranges of 0.1-0.45 mg/mL.

## Introduction

Fungal polysaccharides are biopolymers of monosaccharides units joined by glycosidic bonds, which are either part of fungal cell wall or excreted extracellularly to play roles in cell protection or attachment to surfaces (Giavasis, 2014[7]). In recent years, different polysaccharides with biological activities have been isolated from variety of fungi and characterized. Among these biological activities with favorable impact on functioning of human body, the antitumor effect of fungal polysaccharides have attracted a lot of attention of studies, for the polysaccharides have insignificant side effects in comparison with synthetic agents and thus can be an alternative for tumor therapy. To date, a great deal of efforts have been spent to screen novel fungus for producing polysaccharides with particular action against specific cancer cells. Endophytic fungi, which grow within their plant hosts, can produce secondary metabolite with various biological activities (Stierle and Stierle, 2015[15]; Wang et al., 2011[16]). Therefore, endophytic fungi are considered to be a possible useful source of novel polysaccharides. 

Submerged fermentation of fungus is a widely used technique for producing bioactive exopolysaccharides (EPS). In development of submerged fermentation, culture medium exerts a vital function on the growth of mycelium and the yield of EPS production. To devise optimal medium for maximum EPS production not only needs an investigation on effect of each medium component, but also needs to consider interaction effects between the components. Using single factor method involves a large number of experiments and ignores the interactions between variables. Response surface methodology (RSM) provides an efficient solution for the optimization of process affected by multi-factors. RSM was designed to find optimum conditions of factors and evaluate the effects of factors by building a quadratic regression model based on the results of statistical experimental design (Baş and Boyaci, 2007[3]). It has been successfully used to optimize medium composition for enhancing EPS production from various fungi (Kim et al., 2002[10]; Ma et al., 2014[12]).

In our previous work, the endophytic fungus *Bionectria ochroleuca* M21 was isolated from leaf of guava (*Psidium guajava*), and identified by ITS rDNA sequencing. The objectives of this work were first to investigate the effects of medium components on the production of EPS from M21, and then to optimize the medium composition for maximum EPS production using RSM. Furthermore, EPS from the endophytic fungus was examined for antiproliferative effect against different cancer cell lines. This is the first report of antitumor effect of EPS from an endophytic fugus *B. ochroleuca* M21. The result of this study confirmed endophytic fugus *B. ochroleuca* can be a new source of EPS with potential antitumor activity.

## Materials and Methods

### Materials

The endophytic fungus strain *Bionectria ochroleuca* M21 was isolated from *Psidium guajava*. The strain was grown on potato dextrose agar (PDA) slants at 25 °C for 5 d and maintained on PDA at 4 °C. Human liver HL-7702 cells, liver cancer cells (HepG2), gastric cancer cells (SGC-7901) and colon cancer cells (HT29) were from the cell bank of Shanghai Institute of Cell Biology (Shanghai, China). Cells were grown in RPMI 1640 medium (Life Technologies, Grand Island, NY, USA) supplemented with 10 % foetal bovine serum, 100 U/mL penicillin and 50 μg/mL streptomycin in humidified air with 5 % CO_2_ at 37 °C. 3-(4,5-Dimethylthiazol-2-yl)-2,5-diphenyl tetrazolium bromide (MTT) were purchased from Sigma Chemical Co. (St. Louis, MO, USA). All the other chemicals used in this study were of analytical grade.

### Inoculum preparation and culture conditions

The *B. ochroleuca* M21 of stock culture was inoculated to PDA plate and cultured at 25 °C for 5 days. Three round blocks (6 mm in diameter) were cut from the plate culture and then transferred into 250 mL flask containing 100 mL pre-culture medium with the following composition: 20 g/L glucose, 5 g/L yeast extract, 1 g/L potassium dihydrogen phosphate and 0.5 g/L magnesium sulfate with initial pH6.5. The flasks were then incubated in a rotary shaker incubator with 150 rpm at 25 °C for 3 days. The experiments were carried out in Erlenmeyer flasks with different medium, according to the experimental design, inoculated with the pre-cultures in 8 % (v/v) inoculum level and incubated at 25 °C with 150 rpm rotation for 5 days. The experiments were performed in triplicates. The verification experiment was conducted in a 5L agitated bioreactor containing 3.5L optimal medium under following conditions: temperature 25 °C, inoculum level 8 % (v/v), agitation speed 200 r/min and aeration rate 0.8 vvm.

### Medium optimization

#### Single-factor experiment

Effects of medium components on mycelium biomass and EPS yield were investigated using single-factor experiments. Carbon sources, nitrogen sources, mineral elements and surfactants were tested individually by adding to basal medium while keeping other components of basal medium at a constant level. The basal culture medium was composed as follows: glucose 20 g/L, yeast extract 5g/L, potassium dihydrogen phosphate 1g/L, thiamine 0.05g/L, initial pH 6.5.

### Optimization of EPS production using Central composite design (CCD)

A five-level-four-factors design of CCD was used to optimize medium composition for glucose(X_1_), yeast extract (X_2_), MgSO_4_ (X_3_) and Tween80 (X_4_). The designed experiment consisted of 30 runs including 6 replicates of central point, which were used for the estimation of a pure error sum of squares at the center of the design (Table 1(Tab. 1)). 

The experimental design and levels of medium components were listed in Table 2(Tab. 2). The response value gained from the average of triplicates. The results were fitted into a second-order polynomial equation by a multiple regression technique using Design Expert software (Version 7.0, Stat-Ease Inc., USA).


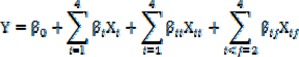


where Y is predicted response (EPS g/L), X_i_ and X_j_ stand for independent variables. β_0_ is the intercept of the regression equation and β_i_ is linear coefficients. β_ii_ is quadratic coefficient and β_ij_ is interaction coefficient.

### Assay of mycelial biomass and EPS content 

Mycelial biomass was expressed as dry cell weight (DCW). Mycelia was separated from sample by centrifugation at 4 °C (6000×g, 15min) and washed twice with distilled water, dried at 60 °C to a constant weight and weighted. The supernatant from centrifugation was filtered through filter paper. The filtrate was mixed with four times volume of ethanol and kept overnight at 4 °C for precipitation. The EPS precipitates were collected by centrifugation (6000×g, 10min), washed three times with ethanol, and lyophilized and stored at -20 °C until analysis. The EPS content was measured by phenol-sulphuric acid method (DuBois et al., 1956[5]) using glucose as the standard.

### Anticancer activity assay

The antiproliferative activity of EPS on the viability of various cancer cell lines and cytotoxicity on human liver HL-7702 cells were determined by MTT assay. Exponentially growing cells were incubated in a 96-well plates at initial density of 1×10^4^ cells/mL for 24 h at 37 °C in a humid atmosphere with 5 % CO_2_. Then the growth medium was replaced with 100 μL medium containing different concentrations of EPS and re-incubated for 24 h for cytotoxicity assay, and for 24 h, 48 h, 72 h for antiproliferative activity assay. Control wells were set with fresh medium, and blank wells contained growth medium without cells. After incubation, the medium was removed and the cells were washed with phosphate buffer saline (PBS). The cells of each well were treated with MTT solution (5 mg/mL in PBS, pH 7.4) at a final concentration of 0.5 mg/ mL and incubated for another 4 h. The supernatant was removed and 150 μL of DMSO added to solubilize the formazan. The plate then was shaken for 15 min and measured absorbance at 490 nm by an Bio-rad xMark™ microplate reader (Bio-Rad Inc., Hercules, CA, USA). Cell viability was calculated using absorbance percent compared to control wells.

### Statistical analysis

The results were expressed as mean ± SD (standard deviation). The data analysis was performed using SPSS 17.0. Differences among treatments were determined by one way ANOVA. The P value less than 0.05 was considered to be statistically significant. The regression analysis of the RSM experimental data, response surface curves and corresponding contour plots were performed using Design expert 7.0 software.

## Results and Discussion

### Effect of medium components on mycelial growth and EPS production

Carbon sources are essential nutrients in culture medium, which play important roles in fungal mycelial growth and metabolite biosynthesis. To identity the suitable carbon for best mycelial growth and EPS production by *B. ochroleuca* M21 different carbon sources were tested. As shown in Table 3(Tab. 3), seven kinds of carbohydrate were employed in the basal medium instead of glucose at a concentration of 30 g/L, respectively. Among tested carbon sources, the maximal mycelial growth was achieved in medium containing glucose, followed by sucrose and maltose. Also, the highest EPS production, 1.87 g/L, was obtained using glucose as carbon source. In contrast, the mycelial growth and EPS production were poor when lactose or sorbitol was used. Nutritional requirements vary much from strains for mycelial growth and EPS production among fungus. It was reported that carbon sources for mycelial growth and EPS production were different. Lactose was found to have best promoting effect on biomass, whereas glucose is the best for EPS production in *Lyophyllum decastes* submerged cultures (Pokhrel and Ohga, 2007[13]). In another investigation on *Shiraia bambusicola*, the highest amount of mycelial was obtained in glucose medium and the maximal EPS was produced in maltose medium (Yang and He, 2008[18]). In the present study, the results indicated that mycelial growth was related to EPS production in a positive correlation manner. Glucose was the most suitable carbon source for both biomass and EPS production, and therefore, glucose was selected as the carbon source in subsequent experiments.

Nitrogen sources support cell growth by providing nitrogen for proteins and nucleic acids synthesis, and also can affect regulatory of enzymes production. A total of nine nitrogen sources, including organic and inorganic nitrogen sources, were tested for screening the best. Each nitrogen source (6 g/L) was added to the basal medium instead of yeast extract. Of all the nitrogen sources tested, yeast extract showed the most advantageous effect on EPS production and growth of mycelial. The maximal biomass and EPS production were achieved with yeast extract after 5 d of cultivation, which reached 7.59 g/L and 1.64 g/L, respectively. This observation might be due to the fact that yeast extract not just contains nitrogen favorable for cell growth and EPS, but also has some vitamins which can be served as vital nutrition factors. From the results listed in Table 3(Tab. 3), it is also seen that organic nitrogen sources were more effective than inorganic nitrogen sources for facilitating mycelial growth and EPS production. The similar observations of better effect of organic nitrogen sources were reported in submerged culture of *Tremella fuciformis* (Cho et al., 2007[4]) and *Cordyceps militaris* (Kim et al., 2003[11]). From the above results, yeast extract was chosen for further experiments to optimize the culture medium for EPS production.

The effect of mineral elements was investigated by adding different mineral sources into the medium at a concentration of 0.5 g/L and no element adding was used as control. In comparison with the control, a higher mycelial growth was achieved in medium with Magnesium ions (Mg^2+^), whereas lower mycelial growth was observed when the medium containing other tested minerals.

In terms of EPS production, Magnesium ions containing medium also have a promoting effect and gained the highest EPS production at 1.86 g/L. Mineral elements are a necessary part of nutrition for mycelial growth and also can function as cofactor of key enzymes for EPS synthesis (Banerjee et al., 2009[2]). In the present study, Magnesium ions showed the best effect on EPS and biomass production, therefore, MgSO_4_ was selected as mineral element for further study. 

Addition of surfactant in submerged culture of fungi had been reported to have stimulating influence on EPS production (Hsieh et al., 2008[9]; Silva et al., 2007[14]). To examine the effect of surfactants in *B. ochroleuca* M21 culture, four kinds of surfactants were added to medium at concentration of 0.1 % (v/v). The results showed that the mycelial growth, when surfactant was added to medium, were not significantly different with control. Among tested surfactants, Tween 80 significantly enhanced EPS production. The highest EPS production (1.90 g/L) was obtained, when 0.1 % (v/v) of Tween80 was used. This corresponds with previous literature reported the stimulating effect of Tween 80 on EPS production in cultures of *Botryosphaeria rhodina* (Silva et al., 2007[14]) and *Schizophyllum commune* (Hao et al., 2010[8]), respectively. The stimulating effect of Tween 80 can be attributed to the stimulation of biosynthetic activity for EPS production and the membrane permeability for EPS secretion (Angelova and Schmauder, 1999[1]).

### Optimization of medium components for EPS production

CCD of Response surface methodology was employed to further investigate the individual and interactive effects of the selected medium components (glucose, yeast extract, MgSO_4_, Tween80) for the production of EPS. Experimental runs with response variable (EPS produciton) were shown in Table 2(Tab. 2). Based on the statistical analysis of the results, a quadratic model was used to express the relationship between EPS production and different medium variables, glucose, yeast extract, MgSO_4_ and Tween80. The equation is given below, in which Y was the predicted response of EPS production, X_1_, X_2_, X_3_ and X_4_ were coded values of glucose, yeast extract, MgSO_4_ and Tween80, respectively.


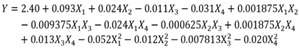


The result of F-test (Table 4(Tab. 4)) indicated that the model was highly significant and suitable to predict the response of EPS production. Goodness-of-fit for the model was evaluated using the R^2 ^value which was 0.9518. This indicated that 95.18 % of the actual value of experimental results corresponded well with the value predicted by the model and only 4.82 % of the total variation remained unexplained by the established model. The high value of F (21.16) and very low probability value (*p* < 0.0001) showed that the model is statistically very significant. The *p*-value (0.6670) for Lack of Fit implies that the Lack of Fit is not significant relative to the pure error, indicating that the proposed model fits well with the experiment results. The lower value of coefficient of variation (1.45 %) demonstrated a greater reliability of the experimental results.

**Figure 1:**
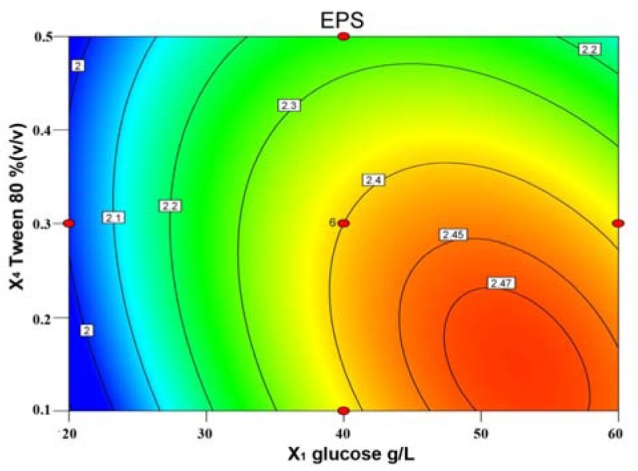
Contour plot of response surface for the effects of glucose (X1) and Tween80(X4) on EPS production

The results of regression analysis showed that the medium components, glucose (X_1_), yeast extract (X_2_) and Tween80 (X_4_) were highly significant factors (*p* < 0.01). Glucose (X_1_) and yeast extract (X_2_) have a positive linear effect on EPS production, and Tween80 has negative effect on EPS production. However, the influence of MgSO_4_ (X_3_) was insignificant (*p* > 0.05) in linear and quadratic terms. The effect of glucose(X_1_) is very important since the highest positive regression coefficient (0.093) was observed for it in the equation. The response of EPS production was also seen affected by the interactive effects between medium components. The *p*-values for X_1_X_4 _was 0.0114 (*p* < 0.05), indicating that the interaction of glucose and Tween80 has significant impact on EPS production. As shown in Figure 1(Fig. 1), a elliptical nature of the contour plots of glucose (X_1_) and Tween80(X_4_) was observed, indicating the interactions between the corresponding variables is significant.

**Figure 2:**
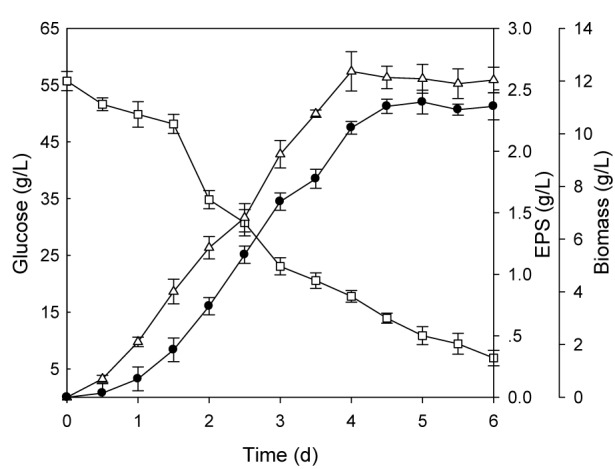
Time course of batch fermentation for EPS production with optimal medium in 5L bioreactor. EPS(△), biomass(●) and glucose(□)

The optimal levels of tested medium components were obtained by solving the regression equation. The predicted maximum EPS production was 2.56 g/L when the following optimal medium was used: glucose 55.7 g/L, yeast extract 6.04 g/L, MgSO_4_ 0.25g/L and Tween80 0.1 % (v/v). The adequacy of the model equation was validated by a total of triplicate verification experiments using optimal medium. The EPS production was 2.59 ± 0.15g/L, indicating the actual value was in agreement with the predicted value. In addition, the optimal medium was further verified by performing batch fermentation of EPS using *B. ochroleuca* M21 in a 5L bioreactor. The EPS production, mycelial biomass and glucose consumption was examined during fermentation (Figure 2(Fig. 2)). The highest concentration of EPS (2.65 ± 0.16 g/L) was achieve after 4 days fermentation using the optimized medium.

### Antitumor effect of EPS from B. ochroleuca M21 in vitro

**Figure 3:**
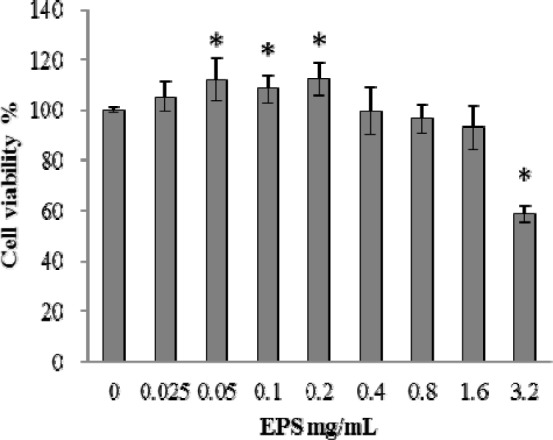
Cytotoxic effect of EPS on human liver HL-7702 cells. Asterisk indicates significant difference from the control (p < 0.05)

**Figure 4:**
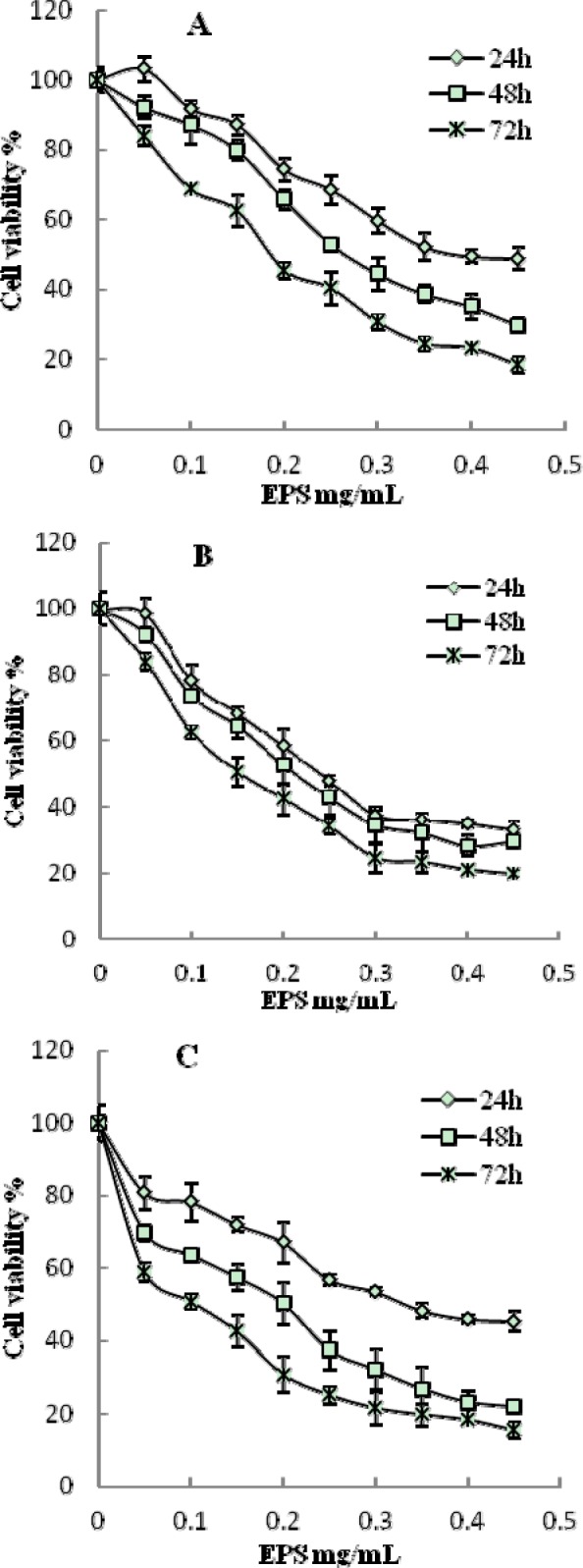
Antiproliferative activity of EPS on the viability of different cancer lines HepG2 (A), SGC-7901 (B) and HT29 (C)

The cytotoxicity of EPS from *B. ochroleuca* M21 on normal cell was investigated by human liver HL-7702 cells. Compared to control, cell viability did not show significant decrease when cells were treated with EPS at concentration 0.025-1.6 mg/mL (*p *> 0.05) (Figure 3(Fig. 3).). The cytotoxicity on HL-7702 cells was only observed at the concentration of 3.2 mg/mL, of which the cell viability was 58.61 %. The ant proliferative activities of EPS on the growth of cancer cells were examined by cell lines of liver cancer (HepG2), gastric cancer (SGC-7901) and colon cancer (HT29). The results showed that the EPS had dose-dependent ant proliferative effects against different cancer cell lines tested in the concentration ranges of 0.1-0.45 mg/mL (Figure 4(Fig. 4).). Also, the time-dependent ant proliferative effects can be seen during incubation time from 24h to 72h, which was indicated by the cell viability of treated cancer cell lines decreased gradually when incubation time increased. After 24 h of treatment, the most significant ant proliferative effect was observed on gastric cancer cells (SGC-7901) at the concentration of 0.45mg/mL among tested cancer cell lines (*p *< 0.05). However, after exposure to the EPS of 0.45 mg/mL for 48 and 72 h, the cell viability of colon cancer cells (HT29) decreased to 21.88 % and 15.42 % respectively, which were significantly lower than that of liver cancer cells (HepG2) and gastric cancer cells (SGC-7901) (*p *< 0.05). MTT assay is an effectively, commonly used method to detect antitumor effect by measuring the cell viability of cancer cells under tested compounds treatment (Furukawa et al., 1991[6]). It had been successfully applied for determining polysaccharide ant proliferative effect on various cancer cells (Ma et al., 2013[12]; Xue et al., 2011[17]; You et al., 2013[19]). The results of the present study clearly demonstrated that EPS from *B. ochroleuca* M21 showed strong ant proliferative activity on three cancer cell lines at tested concentration, and did not exhibit any cytotoxicity on human liver cells at the same concentration. These findings indicated that EPS from* B. ochroleuca* M21 could be used as potential candidate with low negative side effect on normal cells for cancer therapeutics.

## Conclusion

To find optimal medium composition for maximum EPS production from submerge culture of endophytic fungus *Bionectria ochroleuca* M21, different medium components affecting the mycelial growth and EPS production were investigated and optimized by RSM. Glucose was found to be the most significant factor and have a positive effect on EPS production. The optimal composition were glucose (55.7 g/L) , yeast extract (6.04 g/L), MgSO_4_ (0.25 g/L) and Tween80 (0.1 % (v/v)). The maximum EPS production of batch culture in 5L bioreactor using the optimal medium composition was in agreement with the result in flask culture and the predicted value. Furthermore, EPS from *B. ochroleuca* M21 was employed for the first time to investigate its antiproliferative tumor effect in vitro. The EPS showed strong antiproliferative activity on three cancer cell lines and did not exhibit any cytotoxicity on normal liver cell. The results presented in this study provide a useful information for the exploration of EPS as potential antitumor agent from endophytic fungi.

## Acknowledgements

This work was supported by Science and Technology Innovation Project of Education Department of Guangdong Province (2013KJCX0129).

## Figures and Tables

**Table 1:**
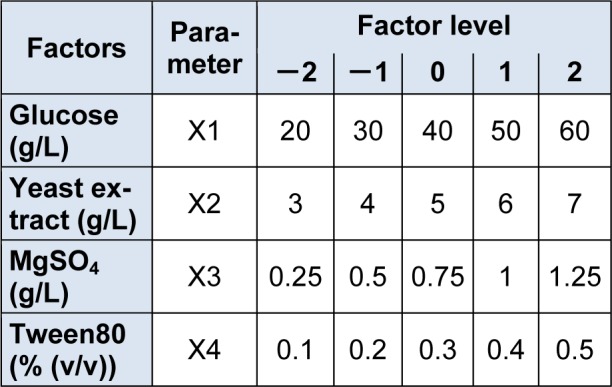
Coded and actual levels of factors in CCD

**Table 2:**
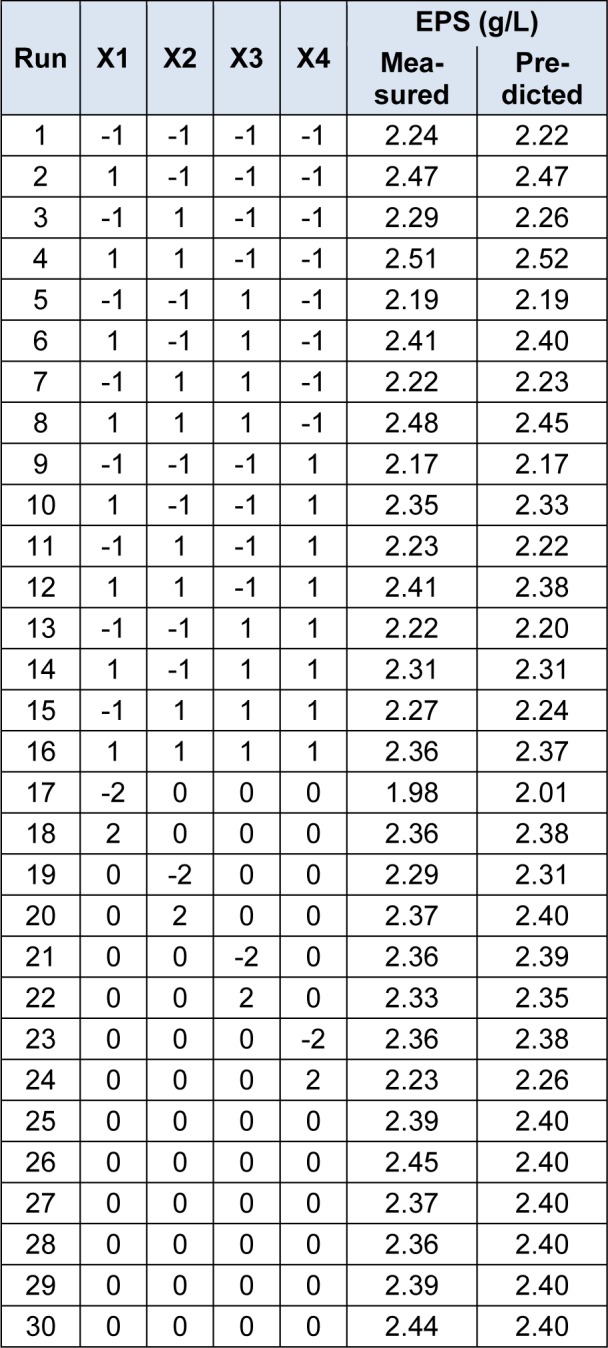
Experiment design and results of CCD

**Table 3:**
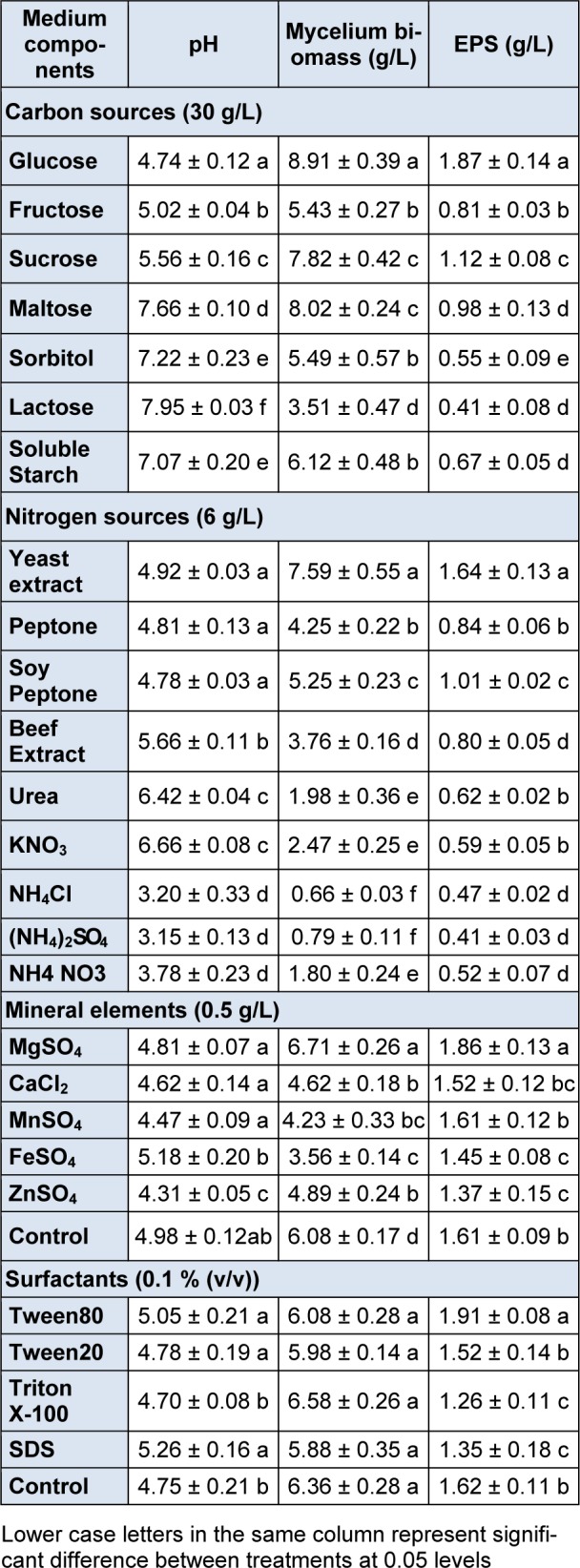
Effect of medium components on mycelial biomass, EPS and IPS yield

**Table 4:**
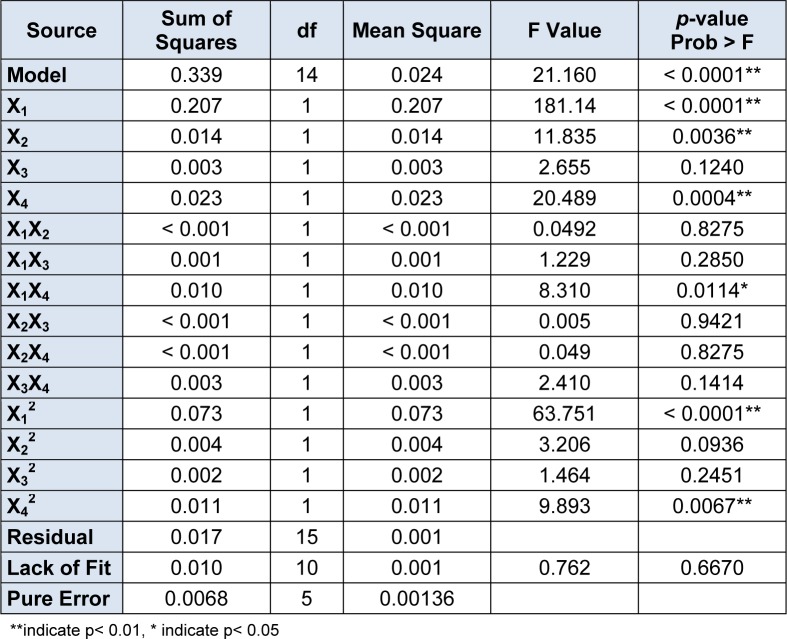
ANOVA for response surface quadratic model
